# Erector spinae plane block did not improve postoperative pain-related outcomes and recovery after video-assisted thoracoscopic surgery : a randomised controlled double-blinded multi-center trial

**DOI:** 10.1186/s12871-024-02544-3

**Published:** 2024-04-23

**Authors:** A. Clairoux, A. Moore, M. Caron-Goudreault, M. Soucy-Proulx, M. Thibault, V. Brulotte, ME. Bélanger, J. Raft, N. Godin, M. Idrissi, J. Desroches, M. Ruel, A. Fortier, P. Richebé

**Affiliations:** 1 Hôpital Maisonneuve-Rosemont, Montréal, Québec Canada; 2 Centre Hospitalier de l’Université de Montréal, Montréal, Québec Canada; 3 Faculté de médecine de l’Université de Montréal, Montréal, Québec Canada; 4 Institut de Cancérologie de Lorraine, Nancy, France; 5 Montreal Health Innovations Coordinating Center, Montréal, Québec Canada

**Keywords:** Thoracic surgery, Video-assisted thoracoscopic surgery, Erector spinae plane block, Plane blocks, Regional anesthesia, Recovery score

## Abstract

**Introduction:**

There is a sizable niche for a minimally invasive analgesic technique that could facilitate ambulatory video-assisted thoracoscopic surgery (VATS). Our study aimed to determine the analgesic potential of a single-shot erector spinae plane (ESP) block for VATS. The primary objective was the total hydromorphone consumption with patient-controlled analgesia (PCA) 24 h after surgery.

**Methods:**

We conducted a randomized, controlled, double-blind study with patients scheduled for VATS in two major university-affiliated hospital centres. We randomized 52 patients into two groups: a single-shot ESP block using bupivacaine or an ESP block with normal saline (control). We administered a preoperative and postoperative (24 h) quality of recovery (QoR-15) questionnaire and assessed postoperative pain using a verbal numerical rating scale (VNRS) score. We evaluated the total standardized intraoperative fentanyl administration, total postoperative hydromorphone consumption (PCA; primary endpoint), and the incidence of adverse effects.

**Results:**

There was no difference in the primary objective, hydromorphone consumption at 24 h (7.6 (4.4) mg for the Bupivacaine group versus 8.1 (4.2) mg for the Control group). Secondary objectives and incidence of adverse events were not different between the two groups at any time during the first 24 h following surgery.

**Conclusion:**

Our multi-centre randomized, controlled, double-blinded study found no advantage of an ESP block over placebo for VATS for opioid consumption, pain, or QoR-15 scores. Further studies are ongoing to establish the benefits of using a denser block (single-shot paravertebral with a continuous ESP block), which may provide a better quality of analgesia.

## Introduction

Thoracic surgery is associated with a high incidence of moderate to severe acute postoperative pain. After surgery, efficient analgesia is paramount to effectively facilitate optimal recovery, increase patient satisfaction, and lower the risk of major postoperative complications [1, 2]. While thoracic epidural analgesia (TEA) is recognized as the gold standard for acute postoperative pain relief after thoracotomy, multiple authors have suggested it is too invasive for video-assisted thoracoscopic surgery (VATS).

Although VATS is associated with decreased tissue trauma and acute thoracic pain compared to thoracotomy, postoperative pain is often still severe. Guidelines for enhanced recovery after lung surgery recommend the use of regional analgesia and opioid-sparing analgesia to facilitate early mobilization and reduce the risk of pulmonary complications. The search continues for the best minimally invasive, low-risk, and effective regional analgesia technique in this population. A systematic review recently concluded that, currently, there is no agreed-upon gold standard [3, 4].

Multiple techniques have been proposed in the literature to fill the research gap on acute pain management after VATS. The paravertebral block (PVB) is often mentioned as it is equivalent to TEA in terms of postoperative and opioid-sparing analgesia after thoracotomy. Furthermore, PVBs have a better side-effect profile (less postoperative nausea and vomiting, pruritis, urinary retention, and hypotensive episodes) [4–6]. However, PVB is still considered an invasive block and holds a questionable benefit-risk ratio for VATS. The duration of a single-shot block is limited, and when using a continuous catheter, the local anesthetic spread is insufficient to cover the numerous implicated dermatomes. PVB is recognized as a neuraxial technique with risks, albeit of spinal hematoma and epidural abscess. Even though PVB and TEA are still used for VATS, there is a sizable niche for a more minimally invasive analgesic technique that could facilitate ambulatory thoracic surgery [2, 7].

The erector spinae plane (ESP) block infiltrates local anesthetics in the fascial plane between the fifth thoracic vertebra (T_5_) transverse process and the erector spinae muscle group. The ultrasound landmarks of the ESP are more easily found and further from the neuraxis and the pleura than the epidural and paravertebral space. This makes it potentially less invasive and provides a more straightforward and safer alternative for VATS. Reports have also shown that this technique can provide anesthesia from T_3_ to T_9_ over the ipsilateral hemithorax [8].

The efficacy of the ESP block for managing severe acute thoracic pain after VATS was unestablished when we started our study’s conception. The literature on the subject mainly consisted of case studies, and, to our knowledge, the ESP block had not been evaluated against a placebo in a randomized controlled study. From this research gap, we designed our multi-centre randomized, controlled, double-blinded study to test our hypothesis that a single-shot ESP block would be superior to placebo in cumulative hydromorphone consumption 24 h after a VATS lung resection.

## Methods

### A. Study design

Our multi-centre randomized, controlled, double-blinded study was approved by the Scientific and Ethics Committee of the Centre intégré universitaire de santé.

et de services sociaux de l’Est-de-l’Île de Montréal associated with the Hôpital Maisonneuve-Rosemont and registered at clinicaltrials.gov (NCT03860480 registred on 21/01/2019). After providing written informed consent, patients undergoing elective VATS at the Hôpital Maisonneuve-Rosemont and the Centre hospitalier de l’Université de Montréal (CHUM) tertiary university hospitals were enrolled from November 2018 to December 2021. We randomized these patients into two groups: bupivacaine 0.5% 30 ml with epinephrine 5 mcg ml^− 1^ (Bupivacaine group) or 30 ml of normal saline (Control group).

Indication for surgery was an elective pulmonary wedge resection, segmentectomy, or lobectomy. All patients were above 18 years old and had an American Society of Anesthesiologists physical status score of I to III. Exclusion criteria were: BMI > 35 kg m^− 2^, chronic pain with regular use of either opioids or gabapentinoids during the 2 weeks before surgery, regular marijuana use, history of thoracic surgery on the same side, anticipated high risk of conversion to thoracotomy, inability to communicate with the investigators, taking anticoagulation or antiplatelet medications (except Aspirin and nonsteroidal anti-inflammatory drugs), suffering from any bleeding disorders, surgery for empyema and sympathectomy, known allergy to local anesthetics or hydromorphone or fentanyl, active infection at the injection site, pre-existing neurological or psychiatric illness, severe cardiovascular disease, liver failure, renal failure (estimated glomerular filtration rate less than 15 ml min^− 1^ per 1.73 m^2^ ), and pregnancy. Patients were also excluded after randomization if they had a perioperative conversion to thoracotomy, severe intra- or postoperative bleeding, required postoperative mechanical ventilation, or a technical failure to proceed with the blocks.

All patients were contacted with a pre-approved script by phone, at our preoperative investigation clinic, or on the day of surgery by one of our attending physicians, an anesthesiology resident, or a research nurse. On the day of the surgery, we obtained written informed consent with written information provided and randomized patients into two groups using computer-generated random numbers and a 1:1 allocation ratio. We completed preoperative QoR-15 questionnaires for all patients. They all received pre-emptive 975 mg oral acetaminophen. A research nurse, otherwise not involved in the care of the randomized patient, prepared the research medication. Identical syringes were used for both groups, blinding the surgeon, the attending anesthesiologist, the research investigators, the nursing staff, and the patient. We maintained blinding throughout the study.

### B. Standardized perioperative protocol

We monitored all patients using a 5-lead electrocardiogram, non-invasive blood pressure, pulse oximetry, end-tidal carbon dioxide, train-of-four stimulation, and a bispectral index device (BIS).

General anesthesia protocol was the same for all patients. We administered all anesthesia and analgesia drugs following an estimation of the adjusted body weight using the Schwartz S.N. formula and followed a standard preoperative fasting protocol. After a 3-min preoxygenation period, we achieved a balanced induction using fentanyl 2 mcg kg^− 1^, propofol 1 to 3 mg kg^− 1^, remifentanil 0 to 1 mcg kg^− 1^, and rocuronium 0.6 to 1 mg kg^− 1^. We used remifentanil to blunt the hemodynamic response to intubation and fentanyl for analgesia during the procedure in relay to remifentanil. At the beginning of the procedure, each patient received standard pre-emptive postoperative nausea and vomiting (PONV) prevention comprising dexamethasone 4 mg IV and ondansetron 4 mg IV at the end.

We intubated and mechanically ventilated all patients using volume-controlled positive-pressure ventilation with a tidal volume of 4–6 ml kg^− 1^ of adjusted weight to maintain end-tidal carbon dioxide tension at 35–45 mm Hg. The lung isolation technique was standardized for all patients with a left double-lumen endotracheal tube.

We maintained anesthesia using sevoflurane and aimed for a BIS of 40 to 60 to a maximum of 1.0 MAC adjusted for the patient’s age with a minimal FiO_2_ to sustain a saturation of 90% or greater. We administered fentanyl doses of 25 mcg IV to maintain blood pressure and heart rate within 20% of baseline values. We paralyzed patients using rocuronium for the surgery and used a train-of-four stimulation throughout the case.

We extubated the patients before transferring them to the post-anesthesia care unit (PACU). The PACU nursing team administered hydromorphone 0.4 mg IV boluses as needed via the PCA pump every 5 min up to 1.2 mg if the pain was more than mild (VNRS score of 4 or more). After that, we instructed patients to use a PCA pump as needed. All hydromorphone doses received were recorded by the PCA pump. PCA settings for both groups were hydromorphone with a bolus of 0.2 mg, lockout time of 5 min, and no background infusion.

### C. Ultrasound-guided ESP blockade

After placing the patient under GA in a standard lateral position, an ESP block expert (≥ 20 ESP blocks completed) performed an ESP block at the T_5_ transverse process using an in-plane approach. ESP block experts performed all blocks in our study. They placed a high-frequency linear probe covered in a sterile sheath in the longitudinal plane 3 cm parasagittal from the midline on the operated side, as defined by Forero et al. 2016 [8]. After standard chlorhexidine disinfection and identification of the transverse process and the trapezius, rhomboid, and erector spinae muscles, the expert inserted an 18 Ga Contiplex Ultra 360 needle in a cephalad-caudad direction until the tip reached the fascial plane deep to the erector spinae muscle. Hydrodissection with normal saline was used to correct and confirm the needle tip position. After confirmation, the expert administered an unidentified solution based on the randomized group and inserted a 20 Ga polyamide catheter up to 3 cm in the identified plane. In the event of conversion to thoracotomy, we excluded the patient from the study and used the catheter postoperatively for up to 4 days with continuous bupivacaine perfusion. If the procedure went as planned, we removed the catheter at the end of the surgical procedure.

### D. Outcome measures

Our primary outcome was the total hydromorphone consumption at 24 h. Secondary outcomes included hydromorphone consumption at 1, 6, 12, and 18 h post-PACU arrival, thorax and shoulder VNRS score at rest and during cough at 1, 6, 12, 18, and 24 h post-PACU arrival, QoR-15 on postoperative-day 1, Ramsay Sedation Scale, pruritus and PONV at the same time points.

Registered nurses recorded total hydromorphone consumption readings from the PCA pumps at different time points after surgery. They also recorded VNRS scores for thorax and shoulder pain at rest and during cough at 1, 6, 12, 18, and 24 h post-PACU arrival and evaluated the PONV score, the Ramsay Sedation Scale, the incidence of PONV, and the incidence of pruritus at the same time points.

The QoR-15 is a patient-reported questionnaire with 15 questions assessing the quality of recovery from anesthesia and surgery. Each question is rated on a scale of 0 to 10, with the highest score being 150 and the lowest being 0. As the score approaches 150, the patient’s recovery is considered improved. The questionnaire evaluates five elements: pain intensity, physical well-being, autonomy, and psychological and emotional states [9]. An anesthesiologist, anesthesiology resident, or research nurse administered the French version of this questionnaire.

### E. Sample size calculation and statistical analysis

Based on a preliminary study at the Hôpital Maisonneuve-Rosemont, the mean consumption of IV hydromorphone was 4.7 (3) mg in the first 24 h after VATS lung resection in the Control group. Therefore, using a two-tailed alpha of 0.05, we calculated that a sample size of 52 patients (26 per group) would provide 80% power to detect a clinically significant decrease of 50% in hydromorphone consumption at 24 h in the Bupivacaine group.

We performed statistical analysis using Prism 7 for Mac OS X, version 7.0d (GraphPad Software, Inc.). The normality of the distribution of hydromorphone consumption was confirmed using the D’Agostino & Pearson normality test. We compared the 24-h total doses of hydromorphone and the consumption of hydromorphone at 1, 6, 12, 18, and 24 h post-surgery with unpaired t-test and expressed as mean (SD). Ramsay Sedation Scale and VNRS and PONV scores were compared using the two-tailed Mann-Whitney test and expressed as median (IQR). Using Fisher’s exact test, we compared the incidence of PONV and pruritus between groups. We used post-hoc repeated measures analysis of variance (ANOVA) to evaluate the effect of the randomized group, time, and health care centre where the surgery was performed (independent variables) on hydromorphone consumption and QoR scores (dependent variables). We also evaluated independent variables for interaction. To control for type-I errors at 5%, we used the Bonferroni correction and a corrected *P*-value for the secondary outcomes. A *P*-value ≤ 0.05 was considered statistically significant.

## Results

**Fig. 1:**
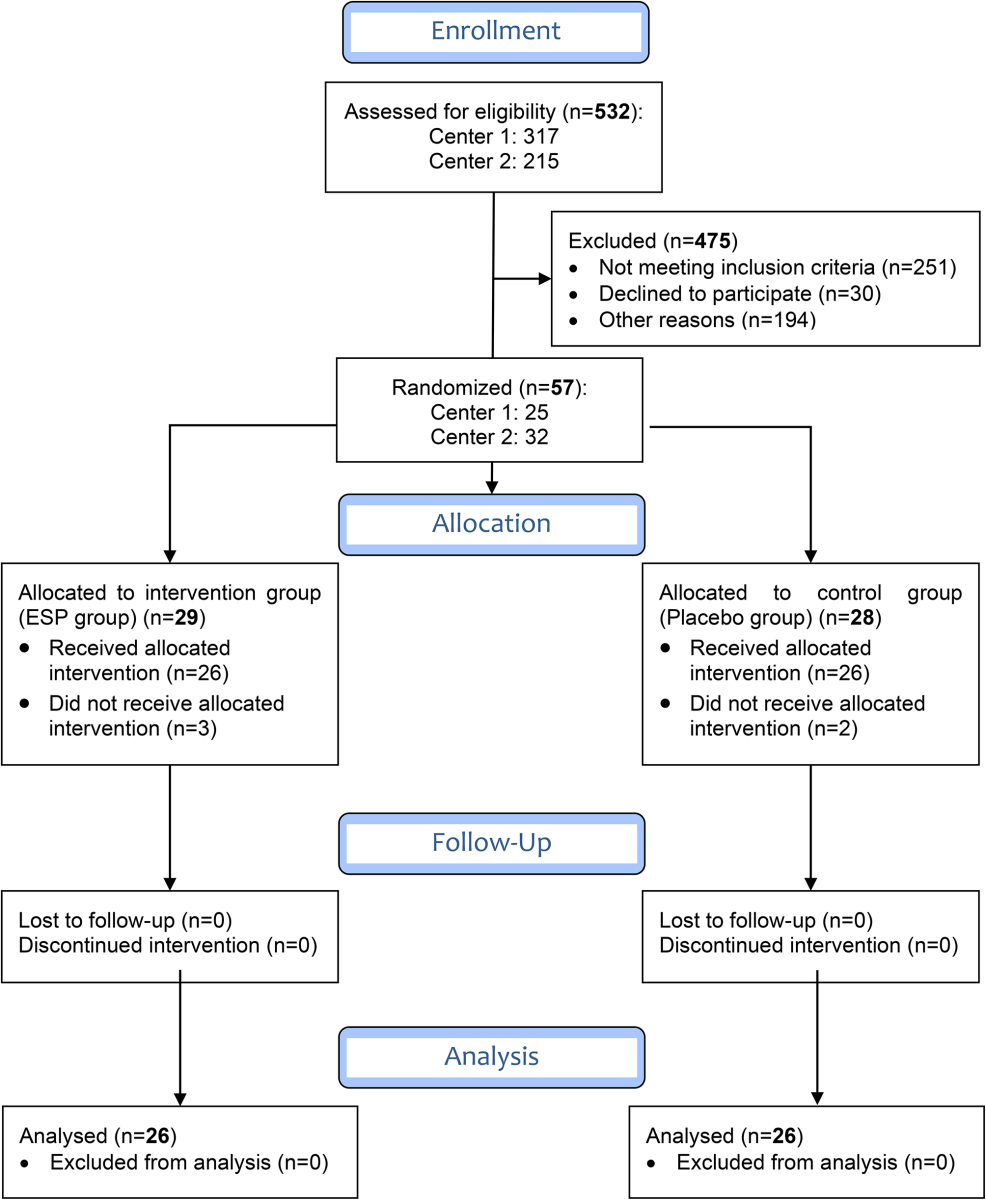
Study flow chart (CONSORT 2010 Flow Diagram). CONSORT indicates CONsolidated Standards Of Reporting Trials ESP = Erector spinae plane

We assessed 532 patients for eligibility, excluded 475 (see Fig. 1 flowchart), and randomized the remaining 57 patients into two groups. A total of 5 patients received the allocated treatment but did not complete the study. In the Bupivacaine group, 1 patient required postoperative intubation, and 2 others needed conversions to thoracotomy. In the Control group, 1 patient needed conversion to thoracotomy, and another had a rescue epidural in the PACU. We excluded these 5 patients from our statistical analysis. Twenty-six patients in each group completed the study, and we analyzed their data. Patient characteristics were similar between the two groups (see Table 1). No other significant complications occurred.

**Table 1:** Demographic characteristics

	Bupivacaine group *N* = 26	Placebo group *N* = 26
Sex: Female, n (%) Male, n (%)	18 (69) 8 (31)	15 (58) 11 (42)
Age (years)	66 (7)	67 (7)
Height (cm)	164 (8)	166 (10)
Real body weight (kg)	73 (12)	76 (15)
BMI (kg/m^2^)	27 (4)	27 (4)
ASA class: 2, n (%) 3, n (%)	19 (73) 7 (27)	15 (58) 11 (42)
Surgery type: Lobectomy, n (%) Segmentectomy, n (%) Wedge, n (%) Biopsy, n (%)	18 (69) 4 (15) 4 (15) 0 (0)	17 (65) 2 (8) 6 (23) 1 (4)

All numbers are represented as mean (standard deviation) or as number of case (percentage)

ASA = American Society of Anesthesiology, BMI = Body mass index

PCA hydromorphone consumption at 24 h after surgery, our primary outcome, was similar between the Bupivacaine and Control groups (7.6 (4.4) vs. 8.1 mg (4.2); *P* = 0.77). There were also no statistical difference between hydromorphone consumption at 1, 6, 12, 18 and 24 h post-operative. Intraoperative fentanyl administration (231 vs. 255 mcg; *P* = 0.55) was also similar. VNRS scores at the thorax and shoulder were evaluated at rest and during cough. Patients were asked what their max pain level was in either situation and what the baseline or mean level was also in either situation. No statistical difference was shown in any of the above mentioned outcomes. (Table 2) PONV scores, the incidence of pruritus and sedation scores were also not different between the two groups at all time points (see Table 2).

**Table 2:** Intra & postoperative data

	Bupivacaine group *N* = 26	Placebo group *N* = 26	Absolute difference (95% CI)	*P* value
Hydromorphone (mg): H1 H6 H12 H18 H24	1.5 (0.8) 3.4 (1.8) 4.7 (2.5) 6.4 (3.5) 7.6 (4.4)	1.7 (0.7) 3.0 (1.3) 4.4 (1.9) 6.0 (2.6) 8.1 (4.2)	-0.2 (-0.6 to 0.2) 0.4 (-0.5 to 1.4) 0.2 (-1.2 to 1.4) 0.4 (-1.4 to 2.2) -0.3 (-2.8 to 2.1)	0.33^b^ 0.36^b^ 0.82^b^ 0.67^b^ 0.77^b^
Fentanyl (mcg)	231 (143)	255 (142)	-24 (-103 to 55)	0.55^**b**^
QoR-15: Preoperative Postoperative Delta QoR-15>=8 Delta QoR-15 < 8	134 (10) 96 (25) 23 (88) 3 (12)	133 (14) 106 (20) 20 (83) 4 (17)	1.4 (-5.5 to 8.3) -11 (-23 to 2) 0.05 (-0.14 to 0.24)	0. 68^b^ 0.10^b^ 0.909^a^
Thorax VNRS at rest: Max Mean	6.0 (2.1) 3.3 (1.5)	5.4 (2.0) 2.7 (1.3)	0.6 (-0.6 to 1.7) 0.6 (-0.2 to 1.4)	0.34^**b**^ 0.12^**b**^
Thorax VNRS at cough: Max Mean	7.1 (2.2) 4.9 (2.1)	6.6 (1.8) 4.6 (2.0)	0.5 (-0.6 to 1.6) 0.4 (-0.8 to 1.5)	0.39^**b**^ 0.54^**b**^
Shoulder VNRS at rest: = 0, n (%) > 0, n (%)	11 (42) 15 (58)	13 (54) 11 (46)	0.12 (-0.16 to 0.39)	0.40^**a**^
Mean	0.8 (1.1)	1.1 (1.4)	0 (0 to 0.8	0.29^d^
Shoulder VNRS at cough: = 0, n (%) > 0, n (%)	11 (42) 15 (58)	14 (58) 10 (42)	0.16 (-0.11 to 0.43)	0.26^**a**^
Mean	0.9 (1.5)	1.4 (1.6)	0 (0 to 1.2)	0.24^d^
PONV score: 1, n (%) 2, n (%) 3, n (%) Mean	14 (54) 9 (35) 3 (12) 1.0 [1.0-1.4]	15 (60) 8 (32) 2 (8) 1.0 [1.0-1.2]	-0.06 (-0.33 to 0.21) 0.03 (-0.23 to 0.28) 0.04 (-0.13 to 0.20) 0 (0 to 0)	0.87^**a**^ 0.54^**d**^
Pruritus score: = 0, n (%) > 0, n (%)	24 (92) 2 (8)	25 (100) 0 (0)	0.08 (-0.08 to 0.25)	0.49^**c**^
Mean	0.02 (0.08)	0.01 (0.04)	0 (0 to 0)	1^d^
RSS score: 2, n (%) 3, n (%) 4, n (%) 5, n (%) Mean	15 (58) 4 (15) 7 (27) 0 (0) 2.1 (0.3)	15 (60) 3 (12) 6 (24) 1 (4) 2.1 (0.2)	-0.02 (-0.29 to 0.25) 0.03 (-0.15 to 0.22) 0.03 (-0.21 to 0.27) -0.04 (-0.12 to 0.04) 0.05 (-0.09 to 0.20)	0.75^**a**^ 0.47^**b**^

All numbers are represented as mean (standard deviation), as median [interquartile range] or as number of case (percentage)

Differences are (Bupivacaine– Placebo):

^**a**^Chi-square test, difference in percentages (95% CI)

^**b**^t-test, difference in means (95% CI)

^**c**^Fisher’s exact test, difference in percentages (exact 95% CI)

^**d**^Mann-Whitney-Wilcoxon test, difference in medians (95% CI) [estimated using the Hodges-Lehmann method]

VNRS = Visual Numeric Rating Scale, H = Hour, RSS = Ramsay Sedation Scale, PONV = PostOperative Nausea and Vomiting, QoR = Quality of Recovery, Max = Maximum

(PONV score: 1 = no nausea, 2 = nausea, 3 = nausea and vomiting)

(Pruritus (itch NRS): 0 = no itch, 1 = itch > 1)

We conducted this study in two health care centres and performed a three-way ANOVA to analyze the effect of the centre, randomized group, and time on cumulative hydromorphone consumption. There was no significant interaction between the different independent variables (group-time-centre (*P* = 0.82), group-centre (*P* = 0.64), time-centre (*P* = 0.10)). Hydromorphone consumption was statistically different at other time points (*P <* 0.001) for both centres and was overall higher at the CHUM centre at any time point (*P <* 0.008); however, the group effect was not significant (*P* = 0.09). Therefore, it is unlikely that the health care centre influenced the difference in hydromorphone consumption between groups (see Table 3).

**Table 3:** Intra & postoperative data by center

	Bupivacaine group *N* = 26	Placebo group *N* = 26	Absolute difference (95% CI)	*P* value
Hydromorphone (mg), H24: Center 1 Center 2 Center 1 & 2	9.0 (4.9) 5.7 (2.7) 7.6 (4.4)	9.3 (4.8) 6.6 (2.8) 8.1 (4.2)	-0.3 (-4 to 3.4) -0.9 (-3.3 to 1.6) -0.3 (-2.8 to 2.1)	0.88^b^ 0.47^b^ 0.77^**d**^
Preoperative QoR-15: Center 1 Center 2 Center 1 & 2	134 (10) 134 (11) 134 (10)	135 (11) 130 (18) 133 (14)	-1 (-10 to 5) 1 (-8 to 21) 1.4 (-5.5 to 8.3)	0.74^d^ 0.9^d^ 0. 68^**a**^
Postoperative QoR-15: Center 1 Center 2 Center 1 & 2	89 (25) 104 (23) 96 (25)	106 (22) 106 (18) 106 (20)	-17 (-36 to 1) -2 (-29 to 17) -11 (-23 to 2)	0.064^b^ 0.86^b^ 0.10^**b**^
Delta QoR-15 > 8: Center 1, n (%) Center 2, n (%) Center 1 & 2, n (%)	14 (93) 9 (82) 23 (88)	11 (85) 9 (82) 20 (83)	0.09 (-0.14 to 0.32) 0 (0 to 0) 0.05 (-0.14 to 0.24)	0.896^a^ 1^a^ 0.9^a^
Delta QoR-15 < 8: Center 1, n (%) Center 2, n (%) Center 1 & 2, n (%)	1 (7) 2 (18) 3 (12)	2 (15) 2 (18) 4 (17)		

All numbers are represented as mean (standard deviation), as median [interquartile range] or as number of case (percentage)

Differences are (Bupivacaine– Placebo):

^**a**^Chi-square test, difference in percentages (95% CI)

^**b**^t-test, difference in means (95% CI)

^**c**^Fisher’s exact test, difference in percentages (exact 95% CI)

^**d**^Mann-Whitney-Wilcoxon test, difference in medians (95% CI) [estimated using the Hodges-Lehmann method]

QoR = Quality of Recovery

We performed a three-way ANOVA to analyze the effect of the centre, randomized group, and time on the QoR-Score. Preoperative QoR scores (134 vs. 133; *P =* 0.68) and postoperative QoR scores (96 vs. 106; *P =* 0.10) did not differ between the groups. There was no statistically significant interaction between the different independent variables (group-time-centre (*P* = 0.41), group-centre (*P* = 0.21), time-centre (*P* = 0.11)). As expected, postoperative QoR-Scores were significantly lower than preoperative scores for both groups (*P <* 0.001). Nevertheless, the randomization into two groups did not show any statistically significant effect on the preoperative or, more importantly the postoperative QoR-Score (*P* = 0.27). Therefore, the evolution of QoR-15 over time was statistically similar for both groups (See Table 3).

## Discussion

Our study showed that an ESP block with bupivacaine 0.5% 30 ml at the T_5_ transverse process administered before VATS lung resection was not superior to normal saline regarding the total hydromorphone consumption 24 h after surgery (7.6 mg vs. 8.1 mg respectively).

Fascial plane blocks are effective and easy to perform, which may explain their recent uptake for postoperative abdominal and breast analgesia. The ESP block is no exception and presents great analgesic potential for multiple surgeries. Enhanced recovery after surgery (ERAS) protocols for thoracic surgeries are becoming more common, and corresponding analgesic regimens must follow ERAS objectives. The ESP block follows these objectives and has some literature to fill that gap. The ESP block is safe and straightforward; the ultrasound target is easily identifiable and distant from neuraxial structures and major nerves or vessels. During an ESP block, a visible cephalad-caudal spread under the erector spinae muscle provides extended coverage on multiple dermatomes. The anterior and posterior thoracic coverage provides postoperative analgesia in the thoracic dermatomes when performed at the T_5_ transverse process and in the abdominal dermatomes when performed lower (T_7_-T_9_). Recent meta-analysis in VATS have also confirmed the superiority in pain management with regional anesthesia techniques such as the ESP block over standard analgesic regimen with benefits in PONV reduction when using regional techniques. [10–12]

Anatomic studies have investigated the local anesthetic spread after an ESP block. Using computer tomography, Bang et al. [13] illustrated that the cephalocaudal spread of a 30 ml injection with contrast at the T_5_ transverse process extends from C_4_ to L_1_. Their findings suggest a potential anesthetic spread to the ventral and dorsal rami on multiple dermatomes. Schwartzmann et al. [14] used magnetic resonance imaging to evaluate the spread of a 30 ml injection at the T_10_ level. Their study identified cephalocaudal spread from T_5_ to T_12_ and transforaminal, paravertebral, and epidural spread. Other anatomic studies have been less conclusive. For example, Ivanusic et al. [15] evaluated the injection spread of 20 ml of dye in 10 cadavers. However, their study did not identify any anterior dye extension and thus could not potential paravertebral, epidural, and ventral rami spread. There was, however, extensive spread laterally and cephalocaudally. Dautzenburg et al. [16] showed unpredictable spread in 11 cadavers, possibly explaining the variability encountered in their clinical practice.

ESP was initially described with excellent analgesic properties in a case series for chronic pain patients. In pediatric case reports, a greater dermatomal coverage was associated with the ESP compared to a thoracic epidural [8]. It proved to be non-inferior to PVB for VATS [17]. In randomized controlled studies, an ESP block confers superior analgesic properties to the serratus anterior plane block [18]. Multiple articles have been published since the introduction of this block concerning its use for cardiac surgery [19–21], abdominal surgery [22, 23], postoperative chronic pain [24], and rib fracture pain [25, 26]. Furthermore, while our recruitment was ongoing, studies were published supporting the superiority of the ESP block over placebo or controls for acute postoperative pain after minimally invasive thoracic surgery in an adult population [27, 28, 29, 30].

A recent randomized study by Ciftci et al. [27] concluded that a single-shot preoperative ESP block with 20 ml of bupivacaine 0.25% led to lower IV PCA fentanyl consumption in the first 24 h after VATS lobectomy compared to a control (no block) group (total consumption of 176 (88) mcg vs. 717 (133) mcg, *P <* 0.001). The authors also reported lower visual analogue scale (VAS) scores and a lower incidence of nausea and pruritus in the ESP group during the first 24 h. While these results are interesting, the internal validity of this study is questionable and may have affected results as only the nurse in charge of evaluating VAS scores was blinded to the allocation group. It is not mentioned if the patients or caregivers were blinded; their control group did not consist of any sham treatment or placebo [27].

In another recent randomized study, Shim et al. [30] concluded that a single-shot preoperative ESP block with 30 ml of ropivacaine 0.5% led to lower numerical rating scale (NRS) scores during the first 6 h after VATS lobectomy compared to a normal saline (placebo) block. Notably, the difference in NRS scores became non-significant 12 h after surgery. The authors also reported a lower incidence of the need for rescue analgesic medication in the PACU, a faster PACU discharge time, and a better Riker Sedation-Agitation Scale score in the ESP group. However, while all patients had an IV PCA fentanyl with a basal infusion of 10 mcg h^− 1^ after surgery and rescue IV meperidine available after PACU discharge, the authors did not report the total consumption. Also, there was a significant difference in NRS scores at 1 and 6 h after surgery, but NRS scores and confidence intervals were not provided. In our study, we could not detect a significant difference between VNRS scores at any time points during the first 24 h; however, we did not design our study to detect such differences.

Additionally, we included VATS patients undergoing less painful procedures than lobectomies, such as wedge resections. These are associated with lower postoperative pain scores and are likely to reduce NRS scores with an ESP block, hindering our ability to detect a difference between the groups. The time-limited analgesic effect of the ESP block described in this study (less than 12 h) may partially explain why we could not find a significant difference in opioid consumption at 12, 18, and 24 h. Additionally, we designed our study to detect a 50% decrease in hydromorphone consumption at 24 h based on similar positive studies. A lower decrease threshold, possibly at a different time point, may have allowed us to detect a clinically and statistically meaningful difference.

Liu et al. [29] recently reported a reduction of approximately 24% in sufentanil consumption at 24 h after a preoperative single-shot ESP block with 25 ml of ropivacaine 0.4% compared with no ESP block (32 (6) mcg vs. 42 (7) mcg, *P <* 0.001). They designed their study to detect a reduction of about 20% in opioid consumption. Intraoperative sufentanil administration, postoperative NRS scores (up to 24 h), the time before first out-of-bed activity, and levels of inflammatory cytokines were also significantly lower in the ESP group. They detected a significant difference in opioid consumption and pain scores up to 24 h after surgery despite recording low pain scores in their control group. This may be attributable to using the less-invasive uniportal VATS approach and the predominant inclusion of wedge resections (about two-thirds of patients).

Finally, Yao et al. [28] reported a higher quality of recovery (QoR-40) score on the day after surgery in patients who received a single-shot preoperative ESP block with 25 ml of ropivacaine 0.5% compared to a normal saline (placebo) block. They also reported a lower IV PCA sufentanil consumption (despite using a background infusion of 2 mcg h^− 1^ in both groups) during the first 24 h after surgery (median 50 mcg, IQR 48–54 versus median 68 mcg, IQR 66–72, *P <* 0.001), lower NRS scores at rest and during cough during the first 8 h, and faster PACU discharge (estimated mean difference of 20 min). While the results of this study are interesting, they used the QoR-40 score; thus, making a direct comparison with the QoR-15 score is unreliable. Again, the total opioid consumption was approximately 25% lower in the ESP group, which was similar to Liu et al. and may further support that our design, based on a reduction of 50% or more in opioid consumption, may have been too restrictive.

Other studies support that the ESP may be non-inferior to a paravertebral block [31, 32]. There is also early evidence that postoperative respiratory function is better with an ESP block than a multilevel intercostal blockade [33] and performs better for VATS than serratus anterior plane blockade [34, 35, 18]. These two techniques are described in the literature as potentially useful for thoracoscopic surgery.

In our multi-centre randomized, controlled, double-blinded study, we could not show a difference in hydromorphone PCA doses or QoR scores in the two groups. Many published case reports initially showed benefits with ESP blocks in specific patient populations (i.e., chronic pain, rib fractures, pectus excavatum) and that the block was comparable to the epidural, a serratus anterior plane block, and intercostal blocks for VATS. However, the first step in implementing a specific block is to compare it against a control, as we have done in our study. When reviewing the literature, remember that all VATS are different; for example, some surgeons could cause minimal tissue trauma while others performed a mini-thoracotomy. It then becomes difficult to compare analgesic needs between such different procedures and, thus, compare study results. The surgeries included in this study were all VATS with minimal incisions and comparable techniques. That might explain why there was no difference between analgesia and QoR-15 scores when performing an ESP block.

We chose a single-shot technique rather than a continuous catheter ESP blockade to establish a potential proof-of-concept for the ESP block as a useful regional technique for future ERAS protocols in thoracic surgeries at our centres. ESP blocks, with the placement of indwelling catheters, have the potential for a longer duration of analgesia by covering multiple dermatomas compared to paravertebral blocks without multiple epidural-associated side effects.

Our study did not entirely implement the suggested ERAS multimodal analgesia regimen [4]. We did not apply nonsteroidal anti-inflammatory drugs to minimize the number of possibly excluded patients because some surgeons believed they would increase the risk of bleeding or surgical anastomosis. We also did not add gabapentinoids to our regimen because it is not widely accepted as a useful perioperative adjunct, especially in elderly patients [4]. To increase the sensitivity of our study, we minimized incremental doses of intraoperative opioids to fentanyl 25 mcg IV at a time.

We administered the blocks pre-emptively to minimize central pain sensitization. In our experience, recovery room pain is lower when plane blocks are performed pre-emptively as they have time to set in. Performing ESP blocks at the end of surgery before extubation might also improve patient satisfaction. However, some studies have found better pain relief when plane blocks were performed 8–12 h postoperatively [36].

## Limitations

Our recruitment of an already rather small sample size was limited by the low capacity to predict the risk of conversion to thoracotomy for some surgeons. A thoracic epidural was mandatory in cases where conversion to a thoracotomy was probable. The Covid-19 pandemic also slowed the recruitment of our study. A significant proportion of screened patients were excluded from the study, often because of the non-availability of ESP block experts or research staff. Patient selection in our study may have impacted the applicability of our results in clinical practice.

Hydromorphone consumption at 24 h in this study was almost two-fold compared to our preliminary data. Unexpected inter-centre variability may have contributed to this finding and may be explained by subtle differences in surgical techniques or postoperative management, such as early mobilization. However, as stated, it is unlikely that the health care centre influenced the difference in hydromorphone consumption between groups. Also, this difference theoretically should have given us more power to detect a clinically significant decrease of 50% in hydromorphone consumption at 24 h in the Bupivacaine group.

At our centre, we have started using single single-shot paravertebral blocks with an added ESP catheter for prolonged postoperative analgesia in our same-day VATS patients. Further studies are needed to evaluate whether this denser primary block (paravertebral block) associated with wider coverage (ESP continuous block) could offer better analgesic results than a single-shot ESP block.

## Conclusion

We could not demonstrate the superiority of the single-shot ESP block compared to placebo for VATS for PCA analgesic consumption nor QoR-15 scores. However, our study’s strength lies in its design; multi-centre (teaching university-affiliated) randomized, controlled, double-blinded study. Our study also included a variety of procedures completed using VATS with minimal tissue trauma, a major factor often not reported in the literature. Our main limitation might have been that we powered this study to show a high difference in opioid consumption (50%). More randomized controlled studies are needed to find the optimal analgesic regimen for VATS.

## Data Availability

The datasets used and/or analysed during the current study are available from the corresponding author on reasonable request.
